# Biosynthesis of Silver Nanoparticles using *Chlorella vulgaris* and Evaluation of the Antibacterial Efficacy Against *Staphylococcus aureus*

**Published:** 2017

**Authors:** Mohammad Soleimani, Maziar Habibi-Pirkoohi

**Affiliations:** Department of Microbiology, Faculty of Medicine, AJA University of Medical Sciences, Tehran, Iran

**Keywords:** Biosynthesis, *Chlorella vulgaris*, Nanoparticles, Silver

## Abstract

**Background:**

It is well documented that Silver Nanoparticles (SNPs) are potent antimicrobial agents. However, little is known about antimicrobial effects of biologically synthesized SNPs at molecular level. In the present study, efficacy of the green microalgae *Chlorella vulgaris* in biosynthesis of silver nanoparticles and inhibitory effect of the biosynthesized SNPs on growth and virulence of *Staphylococcus aureus (S. aureus)* were investigated.

**Methods:**

Algal suspension was incubated in the presence of silver nitrate to induce formation of nanoparticles. The experiment was conducted under a pH range to evaluate pH effect on the shape and properties of nanoparticles. Characterization was performed by Transmission Electron Microscopy (TEM), Scanning Electron Microscopy (SEM), Energy Dispersive Spectrometry (EDS) and X-ray diffraction analysis (XRD). Moreover, concentration of biosynthesized SNPs was measured by high resolution ICP-OES spectrometer. Antibacterial effect of SNPs on growth of *S. aureus* was evaluated by broth micro-dilution method. Inhibitory effect of SNPs on alpha hemolysin, a well-known virulence factor of *S. aureus* was investigated through real time PCR assay.

**Results:**

Spherical SNPs were produced with characteristic monodispersity at low and neutral pHs; however, in alkaline condition, nanorod structures were formed. SNPs inhibited growth of *S. aureus* at concentration of 50 *μg/ml*. Alpha hemolysin expression was also effectively inhibited by SNPs treatment.

**Conclusion:**

In general, results revealed formation of spherical silver nanoparticles with inhibitory effects on bacterial growth and antagonist activity on the expression of alpha hemolysin. Moreover, increase in pH to basic condition resulted in aggregation of nanoparticles and formation of rod-like nanostructures.

## Introduction

Application of metal nanoparticles has increased during recent years in a large variety of fields including electronics, medicine, textile, sensing, *etc* ^1^. The preparation of uniform nano-sized medicine with specific size, shape, and physical and chemical properties is of great importance in formulating a new generation of pharmaceutical products ^2^. It has been reported that nano-based drugs can be used as effective antimicrobial compounds. Therefore, preparation, characterization, modification, and functionalization of metal nanoparticles pave the road for formulation of new bactericidal medicines ^3^.

Promising results have been reported by many authors about the antibacterial properties of various forms of Silver Nanoparticles (SNPs) ^4^. Antimicrobial properties of silver nanoparticles explain the wide application of these particles in various fields of medicine, different industries, animal husbandry, packaging, accessories, cosmetics, health and military ^5^. Silver nanoparticles possess potential antimicrobial effects against a large number of infectious bacteria including *Escherichia coli (E. coli)*, *Bacillus subtilis (B. subtilis)*, *Vibrio cholera (V. cholera)*, *Pseudomonas aeruginosa (P. aeruginosa)*, *Syphilis typhus (S. typhus)*, and *Staphylococcus aureu*s *(S. aureus)* ^3^. Indeed, nanosilver is one of the nanomaterials with the highest degree of commercialization, as roughly 30% of all products currently registered in nano-product databases are claimed to contain nanosilver ^6^.

Although inhibitory effects of SNPs have been widely studied, the majority of the studies have mainly focused on inhibitory effect of SNPs on growth of bacteria and hence, there is a large gap about antagonistic effects of SNPs on virulence factors of the pathogenic microorganisms.

Nanoparticles can be synthesized *via* various methods including mechanical and chemical processes. These methods, though effective in production of metal nanoparticles with various types, suffer from limitations due to environmental and health considerations ^7^. Biosynthesis of nanoparticles using microorganisms, enzymes, and plant extracts has emerged as a clean, cost-effective and efficient alternative to chemical methods. Particularly, biosynthesis of metal nanoparticles by plants is of high potential for commercialization ^8^.

Application of plants and herbal extracts has emerged as a rapid, environmentally safe and cost-effective approach for production of various forms of metal nanoparticles ^9^. During the biosynthesis procedure, functional groups of herbal materials reduce metal ions and play the capping roles as well. Biosynthesis of metal nanoparticles using bioactive compounds found in plant materials (enzymes, proteins, organic acids, and so forth) is a clean and economically affordable approach ^3^. Out of various plant divisions used so far for bioreduction of metal nanoparticles, microalgae appear to be the most ideal platform for nanoparticle synthesis as they grow rapidly and produce large biomass at lower cost ^10^.

There have been a number of studies indicating usefulness of microalgae in biosynthesis of SNPs and other nanoparticles. For example, Annamalai *et al* reported green synthesis of SNPs using aqueous extract of *Chlorella vulgaris (C. vulgaris)* and its inhibitory effect on a number of pathogenic bacteria ^11^. Patel *et al* evaluated efficacy of sixteen macroalga and cyanobacteria species in biosynthesis of SNPs and found out that fourteen species including *Chlorella* sp, *Scenedesmus* sp and *Botryococcus* sp were able to biosynthesize silver nanoparticles with various shapes and sizes ^12^. El-Sheekh and El-Kassas reported *in vivo* biosynthesis of SNPs using *Spirulina platensis*, *C. vulgaris* and *Scene-desmus obliquus*. Furthermore, it was observed that the SNPs biosynthesized by these species had potent cytotoxic activity against *Microcystis aeruginosa* ^13^. In these and other studies, antimicrobial properties of SNPs have been investigated in terms of inhibiting pathogen growth. Therefore, there is a large gap in the literature on inhibitory effects of SNPs on virulence factors of pathogenic microorganisms. This highlights the importance of the present study that aimed at targeting a virulence factor of the human pathogen *S. aureus*.

*S. aureus* is a gram-positive human pathogen that causes serious infections and diseases associated with significant morbidity and mortality. Infection is mediated by a number of virulence factors including several cytolytic, pore-forming toxins. Among the best studied cases of these toxins is the secreted toxin alpha-hemolysin (Hla) ^14^. The present study was conducted to evaluate the biosynthesis of SNPs by the green microalgae *C. vulgaris* and to investigate inhibitory effects of the synthesized SNPs on growth of *S. aureus* and expression of alpha-hemolysin as a major virulence factor of *S. aureus*.

## Materials and Methods

### Chemicals and microorganisms

All chemicals including silver nitrate salt and Luria Bertani (LB) medium were purchased from Merck Inc. (Germany). Primers were chemically synthesized by Macrogen Company (South Korea). cDNA synthesis kit was purchased from Fermentas Inc. (Thermo Fisher Scientific, USA). SYBR green real time PCR master mix was obtained from TakaraBio Inc. (Japan). *C. vulgaris* culture was obtained from laboratory of biotechnology and plant breeding, Ferdowsi University of Mashhad, Iran. *S. aureus* (PTTC 1431) was provided by Mashhad University of Medical Sciences, Iran.

### Biosynthesis of silver nanoparticles

To prepare silver nitrate stock, 3.39 *gr* of AgNO_3_ was dissolved in 100 *ml* distillated water to give a 200 *mM* solution. Healthy culture of *C. vulgaris* was harvested in logarithmic phase and centrifuged at 5000 *rpm* for 15 *min* at 4*°C*. After discarding the supernatant, the biomass was washed with distillated water three times to remove culture medium ingredients. The biomass was re-suspended in 47.5 *ml* of distilled water. Then, 2.5 *ml* of the 200 *mM* AgNO_3_ solution was added to get a final 10 *mM* concentration. A 50 *ml* suspension of *C. vulgaris* free of AgNO_3_ was used as the control. Three pHs as 5, 7 and 11 were used to evaluate acidity effect on SNPs biosynthesis. The experiment was carried out in triplicate and the cultures were incubated at 25*°C* for 48 *hr*.

### Characterization

Bioreduction of silver ions to SNPs was monitored by measuring the UV-Vis spectra of the solution over the range between 300 to 500 *nm* using Spectrophotometer. Morphology of the silver nanoparticles was studied using Transmission Electron Microscopy (TEM) using Leo 912 AB high resolution transmission electron microscope operating at an accelerating voltage of 120 *kV*. A sample of the aqueous biomass solution was placed on the carbon-coated copper grid and dried prior to microscopy. The biosynthesized SNPs were further studied by Scanning Electron Microscopy (SEM) using LEO 1450VT microscope. For XRD measurement, a sample of bioreduction solution was spread in a petri dish and oven dried. The dried sample was taken for XRD analysis using PHILIPS PW1480 X-ray diffractometer. Concentration of biosynthesized SNPs was measured by high resolution ICP-OES spectrometer (SPE-CTRO ARCOS, Germany). For this purpose, a 50 *mg* sample containing both biosynthesized SNPs and algal biomass was diluted and filtered through filter paper and its final volume was adjusted to 25 *ml*. The concentration of SNPs was measured in this 25 *ml* sample.

### Antimicrobial assay

*S. aureus* (PTTC 1431) was provided by Mashhad University of Medical Sciences, Iran. Minimum inhibitory concentration (MIC) of the biologically synthesized SNPs was determined using broth microdilution method in a 96-well standard ELISA plate. Luria Bertani (LB) broth containing 10^5^ *CFU/ml* of *S. aureus* cells was used as a culture medium. The final concentrations of SNPs were 0, 12.5, 25, 50, and 100 *μg/ml*. No SNP was added to negative control well. The lowest concentration of SNPs inhibiting bacterial growth was assigned as MIC. To monitor growth dynamics of bacterial cells during exposure to SNPs, growth curve at 0, 50 and 100 *μg/ml* of SNPs was plotted by measuring optical density (600 *nm*) at different time intervals.

### Real time PCR assay

Real time PCR (RT-PCR) assay was conducted to assess expression of alpha hemolysin (*hla*) under SNPs treatment. RNA isolation and cDNA synthesis were conducted following general procedure. Primer pair sequences for real time PCR of the virulence factor are 5′-TGAATCCTGTCGCTAATG-3′ as the forward primer; and 5′-TATCATCCGACCTTTCACT-3′ as the reverse primer (15). 16s RNA was used as housekeeping (reference) gene and internal control in RT-PCR assay. Forward and reverse sequences of the reference gene were 5′-CGTGCTACAATGGACAATACAAA-3′ and 5′-ATCTACGATTACTAGCGATTCCA-3′, respectively ^15^. Expression of the virulence gene (*hla*) was quantitatively analyzed using a RT-PCR system (BioRad). RT-PCR was carried out in a 20 *μl* reaction volume containing 0.5 *μM* of each primer and 10 *μl* of SYBR green real time PCR master mix (Genet Bio, South Korea). Quantitative RT-PCR experiments were performed in duplicate for each sample. The RT-PCR data were analyzed by the ΔΔCt method as described by Xiang *et al* ^15^.

## Results

### Characterization of SNPs

**Figure 1.**
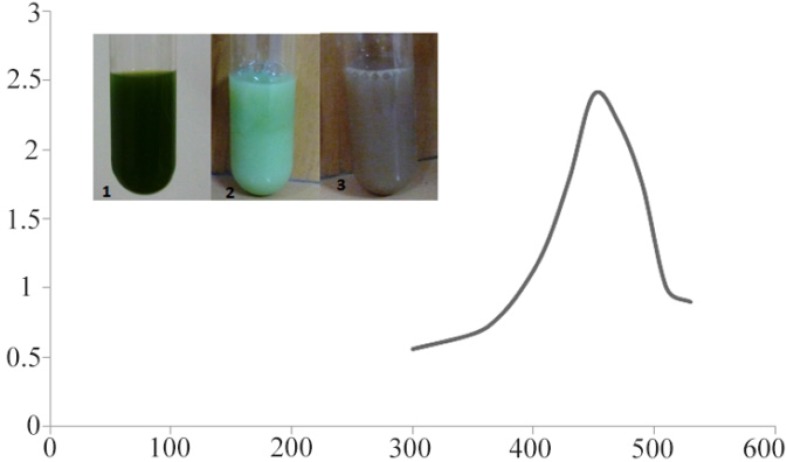
UV-absorption spectra of *C. vulgaris* suspension incubated with AgNO_3_. The absorption peak at 450 *nm* indicates formation of silver nanoparticles. Intersect: gradual color change in *C. vulgaris* suspension indicating bioreduction of silver ions in to nanoparticles.

Color change was immediately observed after adding silver nitrate in to *C. vulgaris* suspension. The dark green color of *C. vulgaris* was changed in to ivory at first and then to brown; indicating biosynthesis of silver nanoparticles. In UV-vis spectroscopy, a surface plasmon resonance peak was observed at about 450 *nm* which confirmed formation of SNPs (Figure 1).

**Figure 2.**
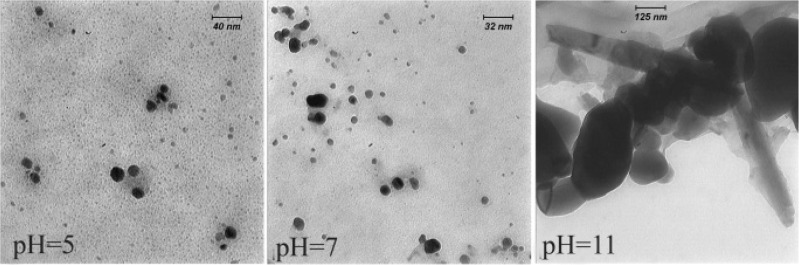
TEM micrograph of biologically synthesized silver nanoparticle at various pHs. Spherical nanoparticles were formed in acidic and neutral pHs; while nanorods were developed at pH=11.

Morphology of the biosynthesized SNPs was revealed by TEM microscopy (Figure 2). TEM image was analyzed using image J software. Based on this analysis, some properties of the biologically synthesized SNPs were revealed that are presented in table 1. In general, TEM microscopy revealed that the silver nanoparticles are of spherical shape with the size of about 10 *nm*. Moreover, most of the SNPs had circularity value as 1, confirming their spherical nature. There was no significant difference between pH=5 and pH=7 regarding morphology of SNPs. However, increasing pH to 11 caused formation of nanorods as depicted in figure 2.

**Table 1.** General properties of the SNPs biosynthesized using *C. vulgaris*

**Parameter**	**Average value**
**Spherical diameter (*nm*)**	10.95
**Width (*nm*)**	6.05
**Length (*nm*)**	7.38
**Roundness**	1.18
**Circularity**	1

**Figure 3.**
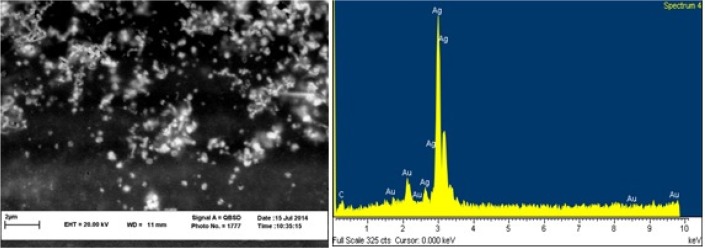
SEM micrograph of silver nanoparticles (left), EDS results indicating sharp peak for silver (Ag^0^).

**Figure 4.**
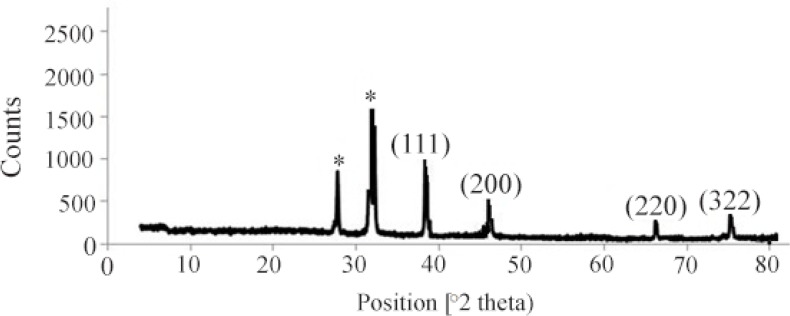
XRD pattern of silver nanoparticles biosynthesized by *C. vulgaris* biomass.

Formation of SNPs was further confirmed by SEM imaging. Energy Dispersive Spectrometry (EDS) study indicated a sharp signal for Ag confirming biosynthesis of silver nanoparticles. An absorption peak at 3 *keV* confirmed presence of silver nanoparticles (Figure 3). Diffraction properties and crystalline structure of the biosynthesized silver nanoparticles were characterized by X-ray powder diffraction. XRD results showed peaks corresponding to (111), (200), (220) and (322) Bragg reflections. This pattern showed presence of SNPs in the sample (Figure 4).

Based on XRD results, particle size can be calculated using Debye-Scherrer formula: d=kλ/βcosθ, Where *d* is the size of nanoparticle, *k* stands for Sherrer constant (0.9), *λ* represents the X-ray wavelength (0.1541 *nm*), β is the Full Width at Half Maximum (FWHM), and *θ* is diffraction angle. Using the Scherrer equation, the average crystallite sizes of the SNPs were found to be in the range of 9–11 *nm*; confirming particle size estimated by TEM images. Concentration of the biosynthesized SNPs was determined using ICP method. Results showed that the concentration of SNPs in a 25 *ml* sample containing both SNPs and algal biomass was 2.934 *mg/l* in average.

### MIC measurement

Serial microdilution was used to evaluate the inhibitory effect of SNPs on growth of *S. aureus*. Results of this test showed that SNPs at the concentration of 50 *μg/mlcan* inhibit growth of the pathogen; the Minimum Inhibitory Concentration (MIC) of the SNPs was therefore determined as 50 *μg/ml*. Bacterial suspension showed normal growth below MIC value. Bacterial growth was also normal at negative control well.

### Growth curve

**Figure 5.**
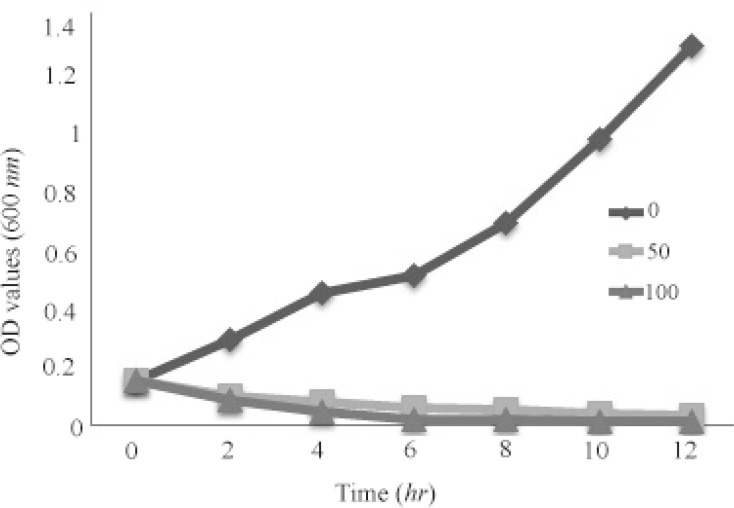
Growth kinetics graph of the bacterium under these SNPs treatments.

To further study the influence of SNPs on *S. aureus,* growth kinetics of *S. aureus* in a 12 *hr* period was monitored under three concentrations of silver nanoparticles as 0 (control, non-treated), 50 *μg/ml* (MIC) and 100 *μg/ml* (the highest concentration in this study). Growth kinetics graph of the bacterium under these SNPs treatments is presented in figure 5. Control group showed a rapid growth pattern which reached its maximum level about 12 *hr* after culture. The bacterial sample treated with 50 *μg/ml* of SNPs manifested a decreasing growth pattern, so that after 10 *hr* the growth level was nearly zero. Growth decreasing was more severe in the sample treated with 100 *μg/ml* of SNPs, so that the growth was observed only after 4 *hr*, and the bacterial growth was completely inhibited 6 *hr* after the treatment.

### Inhibitory effect of SNPs on alpha hemolysin

**Figure 6.**
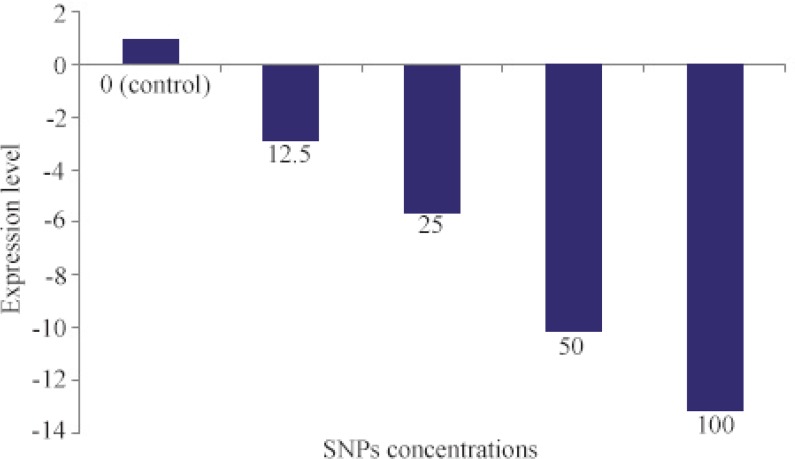
Quantification of alpha hemolysin gene (*hly*) expression in various concentrations of SNPs (*μg/ml*). *: Significantly different from control (p<0.05).

Influence of SNPs on alpha hemolysin-as a virulence factor of *S. aureus*- was studied *via* RT-PCR. Results of RT-PCR are presented in figure 6. As can be seen, a dose-dependent decrease in expression of alpha hemolysin under treatment with various concentrations of SNPs was recorded. In the range of 0 to 100 *μg/ml* of SNPs, a nearly linear relation was observed between SNPs concentration and reduction of alpha hemolysin expression.

## Discussion

By increase of bacterial strains resistant to multiple antibiotics and growing interest on medical costs, many researchers have conducted experiments to develop new, potent and cost effective antimicrobial drugs to address these issues. This scientific movement has led to reinvigoration in formulation of silver-based drugs with broader activity and less probability of inducing microbial resistance than antibiotics ^16^.

Nanobiotechnology has created a link between nanotechnology and biotechnology during recent years. As a branch of nanobiotechnology, biosynthesis of metallic nanoparticle using a wide range of biological agents offers many advantages over conventional chemical and mechanical synthesis procedures as noted earlier in this paper. In the present study, biosynthesis of silver nanoparticles using suspension of *C. vulgaris* was investigated. Microalgae are natural bioremediators that, due to their surface characteristics, accumulate large amount of metal and metallic pollutants. During this bioaccumulation procedure, non-toxic metal containing compounds together with nanoparticles are generated from the trapped metal ions ^17^. *C. vulgaris* is unicellular microalga which naturally occurs in lakes with high level of carbonate and bicarbonate ^18^.

The first indicator of bioreduction of Ag ions to silver nanoparticle is the characteristic change in color of the algal suspension. Some theoretical mechanisms have been proposed to explain biosynthesis of nanoparticles by microalgae. The most probable mechanism is secretion of cellular reductases in to growth medium by microalgal cells. These enzymes can efficiently reduce silver ions in to silver nanoparticles ^19^. Moreover, metal ions can be trapped by the carboxylate groups residing on the surface of microalgal cells. The entrapped ions are then reduced by reductase enzymes which subsequently results in the formation of nanoparticles ^20^. Presence of a maximum peak at about 450 *nm* in UV-vis spectroscopy further confirmed biosynthesis of silver nanoparticles. Surface plasmon resonance peak in the range of 410 *nm* to 450 *nm* has been reported by other authors as an indicator of SNPs biosynthesis ^18,21^.

Morphology of the biosynthesized SNPs was analyzed by TEM microscopy. Biologically synthesized nanoparticles can occur in various forms including rectangular, cubic, spherical, as well as others. TEM images of the present study revealed that the biosynthesized silver nanoparticles have spherical shape with average diameter of 10.95 *nm* (Table1). Moreover, circularity of the biosynthesized SNPs was estimated to be 1, reconfirming spherical shape of the SNPs. Another finding of this research was formation of nanorods at alkaline pH. These structures are expected to be the product of aggregation of individual nanoparticles. It has been reported that at higher pHs, nanoparticles tend to aggregate and form rod- and wire-like structures ^22^.

Presence of SNPs in *C. vulgaris* suspension was further confirmed by SEM images, EDS graph and XRD analysis. An absorption peak at 3 *keV* in EDS study confirmed presence of silver nanoparticles in the solution. The microalgal biomass containing silver nanoparticles was dried and powdered for XRD analysis. Four peaks corresponding to (111), (200), (220) and (322) Bragg reflections were observed in this analysis. The XRD pattern obtained in this study was in accordance with previously determined Bragg reflections associated with silver nanoparticles ^5,23^. Particles size was estimated using XRD data and Debye-Scherrer formula indicated that the biosynthesized SNPs were 9–11 *nm* in average, which agrees with TEM microscopy results.

After characterization of SNPs, their antimicrobial effect on *S. aureus* was studied by serial microdilution method. *In vitro* microdilution test showed that SNPs can inhibit the pathogen growth at concentration of 50 *μg/ml*. Antimicrobial effect of silver nanoparticles has been reported by many authors ^21,24,25^. Silver nanoparticles produced in this study were about 10 *nm* and they were of ideal size for inducing inhibitory effects on bacterial cells. The size of nanoparticles plays a critical role in their efficacy to inhibit microbial growth ^26^. It has been postulated that nanoparticles with smaller sizes have better antimicrobial effect, because they have larger surface area and higher percentage of interaction than bigger particles ^24^. Inhibitory effect of silver nanoparticles on bacterial growth can occur in many ways; for example, silver nanoparticles can interfere with sulfur containing biomolecules residing on the bacterial membrane, or they may attack bacterial genome and respiratory chain. These interfering effects ultimately result in bacterial cell death ^27^.

In addition to investigating inhibitory effect of the SNPs on growth of *S. aureus*, their influence on expression of alpha hemolysin as a major virulence factor of the pathogen was also evaluated. Our results showed that SNPs, even at concentrations below MIC value, can reduce expression level of alpha hemolysin. Alpha hemolysin is an essential factor of lethal pneumonia in a murine model which causes alveolar injury and epithelial barrier disruption ^28^. Negative effect of the biosynthesized SNPs on expression of virulence factors may offer medical implications in developing new antimicrobial medicines. In fact, neutralization of virulence factors is a promising strategy to develop effective antimicrobial agents to combat staphylococcal infection ^28^.

## Conclusion

In this study, the efficacy of green microalgae *C. vulgaris* in biosynthesis of SNPs and inhibitory effects of the SNPs on growth of *S. aureus* and expression of alpha hemolysin were investigated. The results obtained in this study indicated formation of monodisperse SNPs that inhibit growth of *S. aureus*. Moreover, RT-PCR results revealed reduction of *hla* gene under SNP treatment. In general, our study contributes to existing literature on silver nanoparticles by reconfirming applicability of biological systems for rapid, eco-friendly and cost effective production of metal nanoparticles. Antagonistic effect of SNPs on *Hla* gene as a major virulence factor of *S. aureus*- can be viewed as a promising step toward realizing production of SNP-based antimicrobial medicines.
